# Age-Related Epigenetic Derangement upon Reprogramming and Differentiation of Cells from the Elderly

**DOI:** 10.3390/genes9010039

**Published:** 2018-01-16

**Authors:** Francesco Ravaioli, Maria G. Bacalini, Claudio Franceschi, Paolo Garagnani

**Affiliations:** 1 Department of Specialty, Diagnostic and Experimental Medicine (DIMES), Via San Giacomo 12, 40126 Bologna, Italy; francesco.ravaioli2@unibo.it; 2 CIG, Interdepartmental Center ‘L. Galvani’, Alma Mater Studiorum, Via G. Petroni 26, 40126 Bologna, Italy; 3 IRCCS Institute of Neurological Sciences, Via Altura 1-8, 40139 Bologna, Italy; mariagiuli.bacalini2@unibo.it (M.G.B.); claudio.franceschi@unibo.it (C.F.); 4 Karolinska Institute, Clinical Chemistry, Department of Laboratory Medicine (LABMED), H5, Huddinge University Hospital, 14186 Stockholm, Sweden; 5 CNR Institute of Molecular Genetics, Unit of Bologna, 40136 Bologna, Italy; 6 Applied Biomedical Research Center (CRBA), S. Orsola-Malpighi Polyclinic, 40138 Bologna, Italy

**Keywords:** aging, cell reprogramming, induced Pluripotent Stem Cells, DNA methylation

## Abstract

Aging is a complex multi-layered phenomenon. The study of aging in humans is based on the use of biological material from hard-to-gather tissues and highly specific cohorts. The introduction of cell reprogramming techniques posed promising features for medical practice and basic research. Recently, a growing number of studies have been describing the generation of induced pluripotent stem cells (iPSCs) from old or centenarian biologic material. Nonetheless, Reprogramming techniques determine a profound remodelling on cell epigenetic architecture whose extent is still largely debated. Given that cell epigenetic profile changes with age, the study of cell-fate manipulation approaches on cells deriving from old donors or centenarians may provide new insights not only on regenerative features and physiology of these cells, but also on reprogramming-associated and age-related epigenetic derangement.

## 1. Introduction

Due to the increasing life expectancy of the population of industrialised countries and the ever-growing increase in the need for hospitalisation and health care management for the elderly, research in the field of aging has gained in the last few decades a particular relevance within the scientific community. 

Aging is complex phenomenon where genetic, epigenetic, stochastic, and environmental factors intervene in determining a variably quick, progressive physiological decline [1]. Earliest studies on aging and longevity relied on the use of animal models of increasing complexity (from yeast to mice) [2]. Aging studies on human relied on the analysis of large tissue repositories from numerous subjects at different age or repositories of samples from follow up studies. The need for large cohorts of patients at different age or for long-term follow-up studies, and the limited availability for certain tissues made these studies particularly challenging.

The introduction of cell reprogramming techniques granted researchers access to a theoretically unlimited source of hard-to-gather, genetically homogenous cell types and tissues. Cell reprogramming is based on inducing the expression of immature state-specific transcription factors (*OCT4*, *SOX2*, *KLF4*, *c-MYC*, or original Yamanaka reprogramming factors (OSKM)) in adult somatic cells, thus achieving cell fate conversion from a nullipotent, mature state to a pluripotent, embryonic-like one [3]. Early approaches were based on integrating vector-mediated delivery of reprogramming factors (OSKM) that subsequently evolved through the years in order to grant generation of bona fide induced pluripotent stem cells (iPSCs) devoid of any viral and virally inserted components [4,5,6,7].

Induced pluripotent stem cells revolutionized the field of cell-based disease modelling, including age-related pathologic conditions. Indeed, iPSCs have been successfully applied in order to study the onset of several age-related degenerative disorders, such as Alzheimer’s Disease [8], Parkinson Disease (PD) [9,10], and Diabetes Mellitus [11], but also to monitor cellular physiology derangement in accelerated-aging pathologies such as Hutchinson-Gilford progeria [12] and Werner Syndrome [13]. 

Age is intrinsically associated with cell fate and influences the outcome of cell-fate manipulating procedure in two ways: it regulates the ability of the cells to be reprogrammed and it influences the ability of reprogrammed cells to generate fully proficient tissues. Only a limited series of works investigates how age influences reprogramming and how aging trajectories are affected following reprogramming and differentiation. 

In this review, we will summarize existing literature studying the effect of age on cell reprogramming. In addition, we will examine how hallmarks of age are modified by extensive cell fate manipulation with major attention given to DNA methylation (DNAm). Finally, we will get further insights on how age trajectories are altered following reprogramming and differentiation. 

## 2. Effects of Donor’s Age on Cell Reprogramming

Induced pluripotent stem cells have been generated from a large set of different tissues [5] deriving from subjects of different age [14]. Nevertheless, while iPSC-relevant literature overflows with evidence of reprogramming from young-derived cells, the use of elderly-derived tissues seems largely limited. Early experiments that were performed on cells deriving from young and old mice allowed for the derivation of pluripotent stem cells, confirming that reprogramming could affect cells at any age. Using Yamanaka’s factor and a retroviral vector, Kim et al. [15] generated iPSC from 12-month old mice dermal fibroblasts and bone-marrow hematopoietic progenitors at an efficiency (0.02%) five-fold lower when compared to juvenile mice (0.1%). These cells expressed pluripotency-associated markers and were able to differentiate both in vivo and in vitro into all three germ layers, as well as generate CD11b, CD115-positive myeloid cells. Afterwards, Wang [16] successfully reprogrammed 1.5-month (1.5 m), 6-month (6 m), and 14-month (14 m) old mice fibroblasts into iPSC. Reprogramming of 1.5 m and 6 m old mice fibroblasts generated six times more iPSC colonies than 14 m old ones [16]. In addition, Cheng et al. [17] showed that bone marrow cells deriving from two-month old mice could be reprogrammed at a fivefold lower efficiency and twofold slower rate when compared to 23-month old ones.

These studies highlighted a generally lower reprogramming efficiency for slow-replicating, elderly-derived cells when compared to fast-replicating, young-derived ones.

Indeed, the age of cell donor is one of the various factors that have been correlated alongside transfection approach and tissue of origin to reprogramming efficiency of both mouse and human somatic tissues. 

Reprogramming of elderly-derived dermal fibroblasts has been proven to be less efficient when compared to young or neonatal ones. Trokovic et al. [18] observed a negative correlation (r = −0.8916; *p* value = 0.0002) between donor’s age and reprogramming efficiency of dermal fibroblasts transfected with a retroviral OSKM vector. The reported reprogramming efficiencies varied from 0.06% in newborn fibroblasts to 0 in 83-years-old ones. As a further notice, the authors highlight a negative association between reprogramming efficiency and replicative passages thus supporting previously reported evidence suggesting the role of p16/p21-dependent senescence response in determining refractoriness of elderly deriving cells to cell-fate manipulations [19]. 

Despite lower efficiency when compared to young subjects, reprogramming of elderly-derived human fibroblasts into bona fide iPSCs is feasible and has been achieved by multiple research groups, as indicated in Table 1. In one of the earliest attempts, Boulting et al. [20] used a retroviral *OCT4-SOX2-KLF4* (OSK) vector to transduce human fibroblasts gathered from individuals up to 82 years old. iPSC lines generated from both young and old subjects showed pluripotent markers expression and displayed proficiency to differentiate in vitro and in vivo into all three germ layers. 

Reprogramming from elderly-derived tissues was subsequently achieved using different approaches. In 2012, Ohmine [26] reprogrammed keratinocytes from elderly (78 y.o.) Diabetes Mellitus patients into pluripotent stem cells through a lentivirally-delivered OSKM-vector. Furthermore, Wen [24] used a retroviral OSKM-vector to reprogram mucosal fibroblast from a 78 y.o. and 47 y.o. women with pelvic floor disorders. Similarly, Frobel [25] achieved successful iPSC reprogramming from 74 y.o. dermal fibroblasts by retrovirally-delivering the same set reprogramming factors. 

The growing evidence supporting elderly-derived reprogramming feasibility led scientists to start taking into consideration extreme aging phenotypes. In this frame, centenarian biological material started to be considered for reprogramming. Centenarian subjects are unique tools in the study of aging and longevity. Centenarians are, by definition, subjects who largely postponed any major health-threatening, age-related disease. For this characteristic alone, centenarians are considered extremely informative subjects since are the living representative for a healthy, disease-free, aging process [27]. In 2011, Lapasset et al. [22] achieved reprogramming of mitotically inactive cells and fibroblasts deriving from a 101 y.o. subject using a lentiviral vector containing *LIN28*; as well as *OCT3/4*, *SOX2*, and *c-MYC*. Introduction of *LIN28*, which acts by preventing activation of cell maturation and differentiation pathways, as well as *NANOG*, one of pluripotency master regulators, alongside canonical reprogramming factors was a necessary modification in order to grant generation of iPSC lines from senescent and centenarian-derived dermal fibroblasts.

Centenarian-derived cell reprogramming has been replicated only recently. In fact, Yagi et al. [23] managed to achieve full reprogramming of dermal fibroblasts deriving from two subjects, respectively, 106 y.o. and 109 y.o. Yagi used an updated version of Lapasset’s reprogramming method relying on retroviral vector carrying the OSKM. The lack of LIN28 indicates that centenarian fibroblasts do not require transcriptional boosting in order to be reprogrammed other than c-MYC, thus reducing the risk of artefact development.

Further advances in reprogramming methodologies allowed for the generation of iPSC from different centenarian tissue types. One example is represented by the work by Lo Sardo [14] and colleagues who reprogrammed PBMC (Periferal Blood Mononuclear Cells) deriving from subjects ranging from 20 to 100 y.o. using a plasmid-based episomal vector delivering *OCT4*-*SOX2*-*KLF4*-*cMYC*-*LIN28* (OSKML) reprogramming factors. Interestingly, in this case, reprogramming efficiency has not been observed to change with chronological age. 

Understanding the driving cause for longevity-associated phenotype is difficult and requires, as previously mentioned, rather unique and highly relevant cohorts. In these regards, iPSCs generated from centenarian biological material may configure as a possible model for the study of aging in hard-to-gather tissue types.

It is evident though that in order to assess the reliability of iPSC models for the study of aging and age-related conditions, it is first necessary to understand how age-related mechanisms are affected by reprogramming. 

## 3. Epigenetic Remodelling during Reprogramming and Differentiation

Cell reprogramming, have proven that processes involved in determination of cell fate and development are regulated by factors are amenable of external manipulation. Cell reprogramming influences cell fate on multiple levels. It does not promote only morphological mesenchymal-to-epithelial transition, but it also deeply affects cell biochemical, transcriptional, and epigenetic landscape. 

Existing evidence indicates reprogramming techniques exert a strong driving effect on several standard age-associated markers such as telomere length [28]; DNA methylation [29,30]; histone modifications [31]; expression of pro-inflammatory factors [32]; and, cell-cycle arrest [33].

Indeed, it has been shown that iPSC re-express telomerase and possess elongated telomeres [34]; present altered histone modification landscape as well as altered DNAm [35]; lack senescence-associated gene expression [24,26]; and, possess mitochondria with increased energetic output and reactive oxygen species (ROS) resistance [36]. 

Among all of the epigenetics mechanisms, DNAm is the one that in both human and mice [37] showed the best performance as a biomarker of chronological age and in many cases with features of biological age such as age-related diseases and mortality among others. The DNA methylation has played an active role in many functions and processes cellular and tissue differentiation, development, as well as cancerogenic transformation. Whether or not the age-related DNAm changes that occur with age play in the onset of age-related physiological decline, and thus, more broadly, in the aetiology of age-related diseases are still to be understood.

Cell reprogramming exerts a profound remodelling of DNAm profiles. All the somatic adult cells lines possess a specific DNAm landscape that acts as an epigenetic fingerprint being indicative for their type, age, and fate. Most (but not all) of these tissue-specific DNAm signatures are altered to favour the establishment of an human embryonic stem cell-like (hESC) DNAm landscapes (Table 2) [38,39,40,41,42].

Despite this profound DNAm remodelling towards hESC state, cell reprogramming is not able to fully reproduce human hESC DNAm landscape. Whole-genome bisulphite sequencing of Yamanaka factor-derived iPSC, nuclear transfer (NT)-derived stem cells (SCs) with in vitro-fecundation (IVF)-derived hESCs showed that iPSCs carried threefold more aberrant CpG and tenfold more aberrant non-CpG methylation when compared to NT hESCs [40]. In addition, iPSCs were reported to harbour more aberrantly methylated sites at imprinted regions than NT and IVF SCs.

It is evident that iPSCs present an altered epigenetic set-up, at least in terms of DNAm profiling when compared to hESCs. To date, there are no data capable of explaining such differences, and most importantly the possible effects that these different epigenetic profiles could exert in the downstream regulations [44]. Moreover, no data is available to understand whether these differences in terms of DNAm are reproducible and correlated with the age of the source cells.

**Figure 1:**
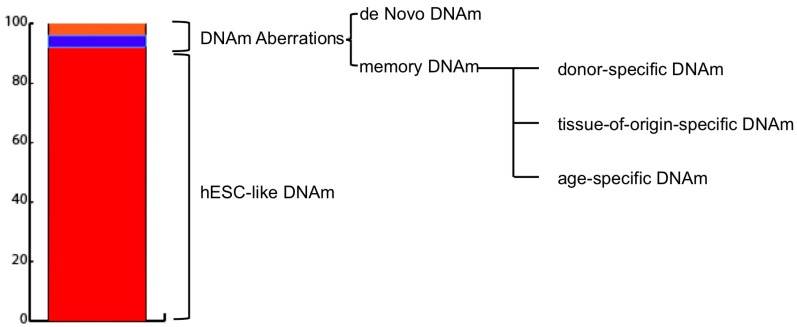
Induced pluripotent stem cells (iPSC) DNA methylation landscape composition. As reported by Lister et al. [39], average DNA methylation (DNAm) values of iPSCs resembles those of embryonic stem cell-like (hESCs). An extremely limited percentage of the sequenced DNA subjected to sodium bisulfite conversion (MethylC-seq) (0.002%) harbours differentially methylated regions (DMRs) (1175 in total). CG-DMRs are subdivided into: De Novo (51–56%) DMRSs which differ both from the parental cell line and hESC and are specific and unique for each iPSC line; and Memory (44–49%) DMRs, which are similar to the parental cell line but not hESC and can be associated either to the parental cell age, type, or could be specific to the parental cell type donor.

Generally, methylation aberrations between reprogrammed cells and hESCs are classified into two main categories: de novo and inherited or memory (Figure 1). The former refers to DNA regions whose methylation levels are significantly different in iPSCs from both parental somatic cell line and hESCs and are specific for each newly-established-iPSC line.

The latter refers to those DNA regions whose methylation levels are similar between iPSC and somatic parental cell and different to hESC one [15,45]. Interestingly, memory DNAm aberrations have been found to provide diagnostic information regarding the cell type [45] and age [14] of the source cells used for reprogramming.

Amplitude of DNAm aberrations is highly inconsistent among different studies. For instance, Lister et al. [39] showed that the vast majority of the differentially methylated regions identified resulted hypomethylated when compared to hESCs [39]; while, Nishino et al. [42] showed that more than 70% of the aberrantly methylated sites were hypermethylated in iPSC as compared to hESCs. 

DNAm aberrations can also occur in genomic imprinted loci [46]. Similar to the above-mentioned cases, DNAm aberrations at imprinted regions appear to be stochastic. Nevertheless, in a study from Bar et al. [47] it was highlighted a specific dynamic of DNAm aberrations occurring at genomic imprinted loci. In details, in this study it was observed that maternally imprinted loci tend to hypomethylation, on the contrary the paternally ones tend to a hypermethylated state. To date, this is the only study that reported such DNAm dynamics and also in this case the aging covariate was not assessed.

Establishment of iPSC aberrant methylation profiles is subject to several variables. Among these, the use of different reprogramming procedures. In fact, comparison of DNAm profiles of iPSCs generated through Yamanaka or Thomson factors (OSKL)) revealed that the majority of aberrantly methylated CpG sites in iPSCs were shared with the parental cell type. Strikingly, reprogramming-method specific DNAm aberrations were identified. In particular, OSKM reprogramming was associated with aberrant hypermethylation; OSKL reprogramming was associated with aberrant hypomethylation [41].

Aberrantly methylated region profiles are not stable and have been found to vary over cell passaging and differentiation. In particular, both inherited and de novo CG-DMRs tend to decrease in amplitude and quantity throughout passaging, ultimately resulting in a partial overlap between iPSC and hESC methylomes [42]. 

Changes in DNAm profile over culturing suggest DNAm establishing mechanisms are active in reprogrammed iPSC cells, thus allowing for further methylome re-shaping and re-differentiation into somatic fate. According to existing literature, differentiation is associated with general reshaping of cell methylome, resulting in hypermethylation of pluripotency-associated regions and hypomethylation of lineage definition-associated ones [48,49]. Differentiation of iPSC into mesenchymal stem cells confirmed the gradual loss of hypermethylation at tissue-specification sites (i.e., *NTE5* and *ENG*) and progressive hypermethylation at pluripotency gene-associated promoter (i.e., *NANOG*; *OCT4*). Nevertheless, the methylome of differentiating iPSC never reached that of somatic adult mesenchymal stem cells (MSCs) even after extensive cell differentiation [25]. Similarly, another study highlighted the fact that DNAm profiles iPSC-derived despite being significantly separated from iPSC-DNAm ones, never reached a similar DNAm profile as parental adult fibroblasts [50]. In another approach, analysis of differentially methylated regions in a model of iPSC-derived dopaminergic neurons revealed that only 35% of neurons-specific differentially methylated regions differed significantly from the undifferentiated state [10].

Incomplete or partial epigenetic differentiation is therefore a commonly observed phenomenon when considering iPSC-based in vitro models. It is not clear whether these differences are associated with limitations in the DNA-methylation establishing machinery or if they are related to a detrimental effect associated with the presence of reprogramming-associated DNAm abnormalities. The fact that a growing body of evidence indicates that iPSC/hESC differentiation efficiency is associated with their DNAm profile indicates that the second hypothesis might be more realistic [51,52,53].

## 4. Epigenetic Age Changes upon Cellular Reprogramming and Differentiation

DNAm patterns are altered with age in all the cell types with directional and stochastic changes. This was observed in humans and mammalian preclinical models [30,54,55]. 

Overall, age-related DNAm alterations follow this general rule: an overall hypomethylation in heterochromatic regions and a gene promoter-specific hypermethylation [56,57]. Of course, these general observations are subjected to a myriad of variants when considering the different genomic regions, cell types, and environmental conditions with a marked inter-individual variability [58,59]. 

Variation in the methylation profile of age-related sites is so robust that several researchers developed tools to measure epigenetic aging. Among the most noticeable examples, Horvath developed an epigenetic aging clock based on a series of 353 CpG sites included in the Infinium HumanMethylation 450 Beadchip (Illumina, San Diego, CA, USA) that allows for accurate age prediction in a large set of healthy heterogenous tissues (PBMCs; whole blood; occipital cortex; breast tissue; buccal epithelium; colon; fat adipose tissue; heart; kidney; liver; muscle; saliva) and individual cell types (CD4 T-cells; CD14 monocytes and immortalized B cells) [55,60]. Horvath’s epigenetic clock has been applied to study epigenetic aging trajectories in age-related health-threatening condition (including Alzheimer’s [61] and PD [62]) in Down Syndrome [63], tumour development [64,65], but also in healthy long-living individuals [66]. In addition, other tools were designed for the assessment of biological aging by analysing CpG methylation at whole genome (Infinium HumanMethylation27 Beadchip, Illumina) [54], or just by focusing on few CpG sites [67]. Among the latters, it is of particular notice the case of the biomarker of biological age based on ELOVL2 CpG island. In fact, this biomarker was not found to be highly correlated with chronological age in blood [68] and dermal fibroblasts [69], but also to highlight association between biological age-acceleration and cancer development [64].

The use of epigenetic-age predicting tools for the study of iPSCs and iPSC-derived cells highlighted a sort of resetting of the epigenetic age. 

Frobel et al. [25] and Lo Sardo et al. [14] studied the reprogramming-associated changes to DNAm age using, respectively, Weidner’s [67] and Horvath’s [55] epigenetic clocks. Both studies reported that the predicted DNAm age of iPSCs deriving from tissues at different age reset from the donor’s epigenetic age to zero. Interestingly, further characterisation of DNAm age dynamics, as provided by Frobel et al. [25], indicated that by differentiating iPSCs back into their parental cell type DNAm age did not increase and only minor changes could be observed upon extensive culturing.

It is worth mentioning that at the moment it is not possible to assess whether the changes in the methylation that is analysed for estimating the biological age produce any relevant biological effect. Accordingly, to date those clocks can be considered just biomarkers. Therefore, any changes that can be observed in any model system cannot be linked to a physiological recovery or acceleration of the aging process. Accordingly, it is necessary to have dedicated studies to investigate whether the reset of the methylation clock that occur in the iPSC generation protocol favour or not also a sort of rejuvenation of the cells. To do it so, it is important that such studies carefully consider the age of the donor of the source cell. 

## 5. Impact of Reprogramming-Associated Alterations in the Study of Age-Related Diseases

**Figure 2:**
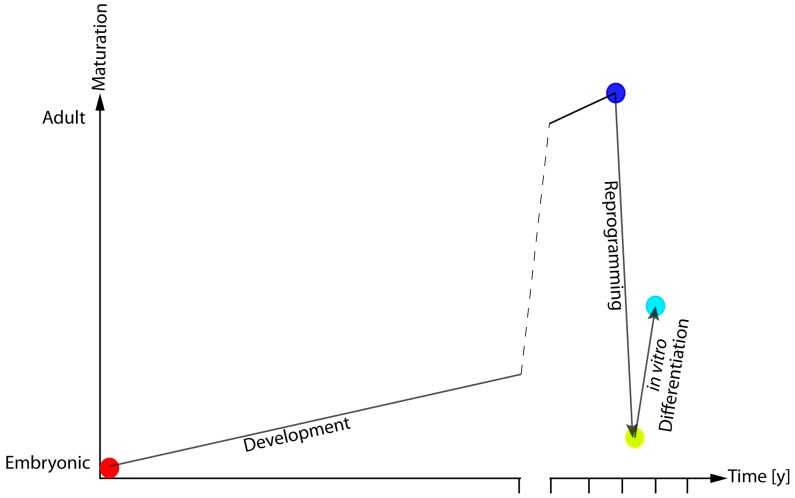
Model of epigenetic and functional rejuvenation hypothesis. During embryonic development, cells undergo a continuous epigenetic and molecular reshaping that ultimately define their function and their anatomical localisation. Cellular reprogramming is a large-scale remodelling procedure that erases most (but not all) of the features defining a cell’s identity. This leads to hESC-like cells that present with altered maturative potential.

The application of the analysis of DNAm pattern in iPSCs and their derivatives indicate that while reprogramming is associated with a reversion of DNAm patterns to embryonic-like state, the differentiation process does not lead to a full re-establishment of the cellular specific DNAm-profile. These results fit with what emerges from recent reports, indicating that tissues differentiated from iPSCs do not present the same physiological and functional features of the target cells rather than they are more similar to an immature version of their adult counterpart (Figure 2) [70,71].

The generation of functionally immature tissues is a limitation for the study of age-related phenomena or late-onset diseases, such as PD, whose specific phenotype is established over the course of several decades. Indeed, PD-relevant iPSC-derived dopaminergic neurons showed only a marginal increase in ROS susceptibility, disease-related gene expression, and α-Synuclein accumulation [72]. In order to gain a profile of disease-relevant characteristics, iPSCs-derived neurons required to undergo in vitro aging through extensive culturing. Indeed, PD-relevant iPSC-derived neurons that were cultured for an extended amount of time showed all disease-relevant characteristics that were lacking or limited in the previous case (high sensitivity to ROS, α-Synuclein accumulation, mitochondria morphological derangement) [72,73]. More recently, Miller et al. [21] proposed a procedure that enhances and accelerates age-related cellular mechanisms in vitro. In this study, for the first time it was successfully applied in vitro cellular age-inducing procedures based on the over-expression of Progerin, a protein over expressed in premature aging syndrome [74] and aged fibroblasts [75].

By inducing acute over-expression of Progerin in young and old donor iPSC-derived dopaminergic neurons, Miller et al. [21] generated cellular models harbouring PD-relevant features (mitochondrial deformation; increased ROS sensitivity; increased neurite degeneration). More recently, Miller et al. published preliminary data suggesting that inhibiting telomerase activity in iPSCs during dopaminergic differentiation exhibited lower survivability, higher DNA damage, and increased ROS production [76]. 

## 6. Conclusions

Over the past few years, interest in the study of aging and age-related pathologies using cellular models, including iPSC increased.

Obviously, cell fate manipulating techniques exert major remodelling over multiple cell physiology-regulating mechanisms, including age-related ones. On the other hand, very little is known regarding the capacity of these stem cells and their derivatives to follow “normal” age-associated mechanics; thus, reliably reproducing the aging process for modelling studies.

In this review, we reported existing evidence linking iPSCs and aging studies in order to understand how age-related mechanisms influence and are influenced by cell fate manipulating techniques. We described how aging is associated with cell capacity to reprogram into pluripotent state. Subsequently, we summarized existing literature reporting changes in age-related biochemical and epigenetic markers, with particular attention to DNAm profile. Reprogramming-associated alterations in cell physiology have been generally associated with the loss of age-related phenotype and acquisition of an embryonic, undifferentiated state. In addition, whether reprogramming is largely associated with reversion of adult-associated DNAm profile towards and embryonic-like state, several methylation aberrations have been highlighted. De novo aberrations are independent to both parental and embryonic DNAm profile, while memory aberrations are maintained from the specific-parental cell DNAm landscape. Among the factors that are involved in the determination of parental cell-specific DNAm profile is age. It is nowadays evident that DNAm changes with age. Age-associated DNAm changes are robust within the population and contribute determining overall aging and health status.

Therefore, understanding how age-specific DNAm mechanics are reproduced in iPSC models is fundamental, although largely neglected by existing literature.

The work we cited by Frobel et al. [25], is the first to apply a biological age-predicting tool for the understanding of the DNAm age dynamics upon differentiation and in vitro aging of iPSC-derived MSCs. The evidence they reported indicates that DNAm age is erased upon reprogramming and is not re-established upon re-differentiation. 

As already mentioned, existing data do not allow for drawing a clear association between age-associated DNAm profiles and any age-related decline feature. Accordingly, it remains still unclear whether erasure age-related DNAm signatures upon reprogramming is functionally associated with physiological alteration of the overall age-related status of the cell. Additional concern can be drawn considering the reliability of existing DNAm age predicting tools. Indeed, Horvath [55], Hannum [54], and Weidner [67] epigenetic clocks are designed based on adult tissues differing tens of years of age. Therefore, they might not be able to grasp DNAm age differences over iPSC-derived, embryo-like differentiated cells and further validation has to be performed on this model.

It is evident that cell reprogramming and differentiation exert profound, stochastic changes whose effect over cell-fate regulating approaches is unclear. Future studies are required to address whether these changes are associated with a functional rejuvenation of the cells. Therefore, it will be critical to thoroughly consider and assess the age of the donor of the source cells and determine whether at different age corresponds also a different epigenetic and functional profile. Understanding this will provide critical insight for considering the use of iPSC deriving from donors at different age for the treatment and the modelling of major age-related health threatening disorders. 

## Figures and Tables

**Table 1:** List of works achieving reprogramming of old- or centenarian-derived tissues.

Reference	Species	Age of Donor	Tissue	Vector	Factors	Efficiency
Kim et al. [15]	Mouse	Juvenile and 12 m	DF; BM	Retroviral	OSKM	5 times higher in juvenile than 12 m old
Cheng et al. [17]	Mouse	2 m and 23 m	BM	Retroviral	OSKM	5 times higher and 2 times faster in 2 m than 23 m old
Wang et al. [16]	Mouse	1.5 m; 6 m and 14 m	DF	Retroviral	OSKM	6 times higher in 1.5 m and 6 m than 14 m old
Miller et al. [21]	Human	11 y.o.; 31–55 y.o.; 71–96 y.o.	DF	Sendai	OSKM	Not assessed
Lapasset et al. [22]	Human	92 y.o.; 94 y.o.; 96 y.o.; 101 y.o.	DF	Lentiviral	OSKMNL	Not assessed
Yagi et al. [23]	Human	106 y.o.	DF	Retroviral	OSKM	Not assessed
Wen et al. [24]	Human	47 y.o. and 78 y.o.	DF	Lentiviral	OSKM	1.3 times more fully reprogrammed lines from 47 y.o. compared to 78 y.o.
Boulting et al. [20]	Human	29–82 y.o.	DF	Retroviral	OSK	Not assessed
Frobel et al. [25]	Human	56 y.o.; 63 y.o.; 74 y.o.	MSC	Retroviral	OSKM	Not assessed
Ohmine et al. [26]	Human	56–78 y.o.	Keratinocytes	Lentiviral	OSKM	Not assessed
Trokovic et al. [18]	Human	0-83 y.o.	DF	Retroviral	OSKM	Negative correlation between donor’s age and reprogramming efficiency (r = −0.89; *p* value = 0.0002)
Lo Sardo et al. [14]	Human	20–100 y.o.	PBMCs	Plasmid + Electroporation	OSKL	No differences in reprogramming efficiency were noticed with increasing age

Age is reported either in months (m) or years (y.o.); DF: dermal fibroblasts; BM: bone marrow cells; MSC: mesenchymal stem cells; PBMCs: Periferal Blood Mononuclear Cells; O: *OCT4*; S: *SOX2*; K: *KLF4*; M: *c-MYC*; N: *NANOG*; L: *LIN28*.

**Table 2:** Studies reporting DNA methylatation (DNAm) changes upon reprogramming.

Reference	Species	Tissues	Transfection Vector	Reprogramming Factors	Methylation Analysis Technique
Ma et al. [40]	Human	FF	Sendai virus	OSKM	Infinium HumanMethylation450 Illumina
Lister et al. [39]	Human	ADS	Retrovirus	OSKM	Methyl-C Seq
Nishino et al. [42]	Human	FLF; AM; E; PDE; MB	Retrovirus	OSKM	Infinium HumanMethylation27 Illumina
Planello et al. [41]	Human	FF	Retrovirus	OSKM/OSKL	Infinium HumanMethylation450 Illumina
He et al. [38]	Human	FF; AF	Lentivirus	OSKM	Infinium HumanMethylation450 Illumina
			Episomal	OSKMNL	
Frobel et al. [25]	HUMAN	BM-MSC	Retrovirus	OSKM	Infinium HumanMethylation450 Illumina
Shao et al. [43]	Human	MSC	Retrovirus	OSKM	Infinium HumanMethylation450 Illumina
Lo Sardo et al. [14]	HUMAN	PBMCs	eD_Plasmid	OSKL	Infinium HumanMethylation450 Illumina

GSC: Germline Stem Cells; FF: fetal fibroblasts; ADS: Adipose tissue-derived Stem Cells; FLF: Fetal Lung Fibroblasts; AM: Amniotic Fluid Cells; E: Endometrium; PDE: Placenta-Derived Epithelium; MB: Menstrual Blood Cells; BM-MSC: Bone Marrow Mesenchymal Stem Cells. eD_Plasmid: electroporated DNA plasmid.
